# Effects of Selenium and Vitamin E on Enzymatic, Biochemical, and Immunological Biomarkers in *Galleria mellonella* L

**DOI:** 10.1038/s41598-020-67072-9

**Published:** 2020-06-19

**Authors:** Mustafa Coskun, Tamer Kayis, Emre Gulsu, Emel ALP

**Affiliations:** Department of Biology, Faculty of Science-Literature, Adıyaman University, Adiyaman, Turkey

**Keywords:** Animal physiology, Entomology

## Abstract

To understand the effects of micronutrients have particular biological functions that are involved mainly in the antioxidant system, which has essential implications for the development of diseases, this study investigated how vitamin E, selenium, and their combination affect lipid, protein, carbohydrate, and malondialdehyde (MDA) content; antioxidant enzyme (catalase [CAT], superoxide dismutase [SOD], glutathione-S-transferase [GST]) activity; and the total hemocyte count (THC) in larvae of *Galleria mellonella* L. fed different diets. Diet 1 (100 µg of selenium) significantly decreased carbohydrate and lipid content. Diets 2 (100 µg of vitamin E), 3 (100 µg of selenium and vitamin E each), and 5 (Tween 80) did not significantly affect protein and carbohydrate content. Diet 2 significantly increased the lipid content compared to diet 4 (control). Diet 1 increased CAT, SOD, and GST activity and MDA content (highest at 27.64 nmol/mg protein). Diet 2 significantly decreased SOD activity and MDA content compared to other diets. Diet 1 significantly decreased the THC compared to other diets. These results suggested that selenium changes oxidative stress parameters, energy reserves, and THC in *G. mellonella*. These changes could be a physiological adaptation against selenium-induced oxidative stress. Vitamin E could play a protective role in selenium toxicity.

## Introduction

Trace elements play a significant role in many biochemical and physiological processes and therefore are essential for living organisms^1^. Selenium (symbol Se), a trace element that is a major component of glutathione peroxidase, plays an important role in intracellular defense against reactive oxygen species (ROS), in addition to being essential for growth and development^2^. However, high selenium levels can be toxic^3,4^. The utility and toxicity of selenium depend on its chemical form and concentration; in its inorganic form (selenates, selenides, and selenite), selenium is more toxic and less useful compared to its organic forms (selenomethionine and selenocysteine). Excessive selenium levels have cytotoxic, genotoxic, and carcinogenetic effects due to ROS accumulation and increased oxidative stress^4,5^.

Cells use antioxidants to neutralize the harmful effects of ROS. Many of these antioxidants decrease DNA damage, decrease protein denaturation or loss, and prevent lipid peroxidation^6,7^. In insects, ascorbate peroxidase, superoxide dismutase (SOD), catalase (CAT), peroxidase, glutathione-S-transferase (GST), ascorbic acid, and vitamin E are the most important components of the antioxidant defense mechanism^6^. SOD is involved in the conversion of the superoxide radical (O_2_^−^) to H_2_O_2_, and the H_2_O_2_ is eliminated by CAT and ascorbate peroxidase. GST is involved in eliminating H_2_O_2_ from cells and lipid peroxidation agents^7,8^ and also decreases the effects of toxins by binding xenobiotics to glutathione (GSH: γ-glutamyl-cysteinyl-glycine). GST is a member of the multifunctional phase II biotransformation multigene enzyme family that catalyzes the conjugation between compounds that carry GSH and electrophilic groups^9,10^. In insects, GST metabolizes many compounds in pathogens, carcinogens, and pesticides and also heavy metals (from environmental pollutants to drugs)^10^. α-tocopherol, the most effective form of vitamin E, has a chain-breaking effect against oxidation of polyunsaturated fatty acids of biological membranes^11^.

The total carbohydrate, lipid, and protein content of diet plays a critical role in the reproductive potential and expansion of the insect population^12,13^. Glycogen is the main source of energy in insects^14^, and studies have reported that changes in glycogen levels are an important indicator of pollution^15^. Lagadic *et al*^15^. further highlighted the importance of this glycogen. Similar to animals, insects need proteins for vital activities, such as development, growth, and reproduction^14^. In addition, amino acids, the building blocks of proteins, play a significant role in neural transmission, phospholipid synthesis, energy procurement, and morphogenesis^16^. In insects, lipids also play an important role in sexual maturity, egg production^12^, and energy provision^17^. Lipids are used as energy sources in detoxification; therefore, a change in their content is considered an important biomarker.

In insects, hemocytes play a significant role in immune reactions (phagocytosis, nodule and capsule formation), transport of materials (food and hormones), and detoxification of xenobiotics (e.g., pesticides and heavy metals). The number and structure of hemocytes could change under xenobiotic stress, and this change is frequently used as a bioindicator for evaluating the effects of environmental contaminants^18,19^.

*Galleria mellonella* L., a moth, serves as a good physiological model to understand the effects of toxic materials on living organisms in nature. In addition, *G. mellonella* is used as a model in physiological, immunological, biochemical, and parasitological studies because of its rapid life cycle, larval size, and easy growth on artificial diets^7,20^.

The use of *G. mellonella* as a model has increased both in studies that evaluate the effects of selenium and vitamin E and in studies that investigate the effects of heavy metals, insecticides, antibiotics, bacteria, fungi, and temperature. Investigating in detail the effects of selenium and vitamin E on *G. mellonella* will contribute to future multidisciplinary studies.

Therefore, this study analyzed the effects of selenium and vitamin E on protein, lipid, carbohydrate, and malondialdehyde (MDA) content; SOD, CAT, and GST activity; and the total hemocyte count (THC) in *G. mellonella*, which was used as a host for the production of many parasitoids in the laboratory.

## Results

### Antioxidant enzyme activity and MDA content

Table 1 shows the effects of diets 1–5 on SOD, CAT, and GST activity and MDA content of *G. mellonella* larvae.

**Table 1:** Effects of diets 1–5 on SOD, CAT, and GST activity and MDA content of *Galleria mellonella* L. larvae.

Diet	Diet content	SOD (U/mg protein)	CAT (U/mg protein)	MDA (nmol/mg protein)	GST (nmol/mg protein)
Diet 1	Selenium	6.44 ± 0.11 a	0.65 ± 0.005 a	27.66 ± 0.60 a	13.38 ± 0.33 a
Diet 2	Vitamin E	4.41 ± 0.10 d	0.44 ± 0.007 c	9.88 ± 0.23 d	9.80 ± 0.39 b
Diet 3	Selenium + Vitamin E	5.82 ± 0.30 c	0.55 ± 0.008 b	13.89 ± 0.28 b	14.12 ± 0.08 a
Diet 4	Control	4.99 ± 0.51 b	0.44 ± 0.011 c	11.19 ± 0.17 c	9.59 ± 0.42 b
Diet 5	Tween 80	5.02 ± 0.08 b	0.35 ± 0.057 c	10.97 ± 0.21 c	10.15 ± 0.81 b

*Student–Newman–Keuls test: The letters a, b, c, and d are used to indicate differences between concentrations. There was a statistically significant difference between data indicated by the same letters (*P* < 0.05).

SOD, superoxide dismutase; CAT, catalase; GST, glutathione-S-transferase; MDA, malondialdehyde.

Diet 1 significantly increased SOD activity compared to diet 4 (*P* < 0.05), while diet 3 significantly decreased SOD activity compared to diet 1 (*P* < 0.05). The lowest SOD activity was observed with diet 2. Diet 1 significantly increased CAT activity compared to other diets (*P* < 0.05). The highest (27.66 nmol/mg protein) and lowest (9.88 nmol/mg protein) MDA content was observed with diets 1 and 2, respectively; there was no significant difference when compared to other diets.

Diets 1, 2, 3, and 5 significantly affected GST activity compared to diet 4. Diets 1 and 3 increased GST activity compared to other diets (*P* < 0.05). Diet 2 had no effect on GST activity (9.80 nmol/mg protein) compared to diet 4; with diet 3, GST activity was 14.12 nmol/mg protein, which was the highest among all experimental groups.

### Protein, lipid, and carbohydrate content

Table 2 shows the effects of diets 1–5 on the protein, carbohydrate, and lipid content of *G. mellonella* larvae.

**Table 2:** Effects of diets 1–5 on protein, carbohydrate, and lipid content of *Galleria mellonella* larvae.

Diet	Diet content	Protein (mg)	Lipid (mg)	Carbohydrate (mg)
Diet 1	Selenium	3.98 ± 0.07 a	7.61 ± 0.14 d	9.34 ± 0.18 a
Diet 2	Vitamin E	4.57 ± 0.08 a	11.24 ± 0.16 a	12.36 ± 0.63 b
Diet 3	Selenium + Vitamin E	3.89 ± 0.34 a	9.40 ± 0.55 bc	11.36 ± 0.33 b
Diet 4	Control	4.43 ± 0.08 a	10.17 ± 0.16 b	11.80 ± 0.32 b
Diet 5	Tween 80	4.48 ± 0.21 a	9.08 ± 0.17 c	10.99 ± 0.66 b

*Student–Newman–Keuls test: The letters a, b, c, and d are used to indicate differences between concentrations. There was a statistical difference between data indicated by the same letters (*P* < 0.05).

The mean protein content per individual was 3.98, 3.89, and 4.43 mg with diets 1, 3, and 4, respectively. Diet 1 tended to decrease the mean lipid and carbohydrate content. Diet 1 significantly decreased the lipid content compared to other diets (*P* < 0.05). The highest lipid content (11.24 mg) was found in larvae fed diet 2. The lowest carbohydrate content (9.34 mg) was found in larvae fed diet 1. Other diets didn’t have any positive or negative effect on the lipid content compared to diet 4.

### Total hemocyte content

**Figure 1:**
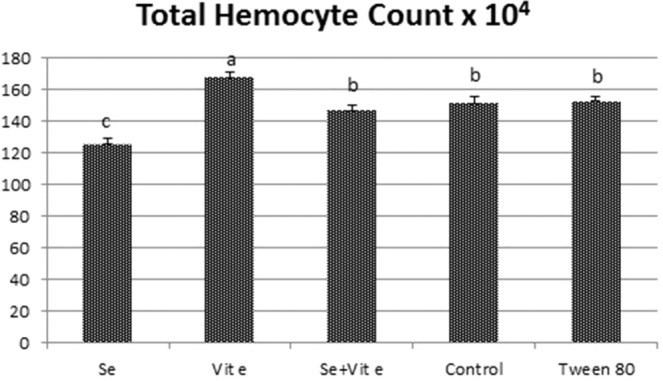
Effects of selenium, vitamin E, and their combination on the THC. THC, total hemocyte count.

Diet 1 significantly decreased the THC compared to other diets (*P* < 0.05), while diet 2 increased the THC compared to other diets (*P* < 0.05). Diet 3 took the THC to the control group’s level. There was no difference in the THC between diets 4 and 5 (Fig. 1).

## Discussion

Toxic substances disrupt the oxidative balance and the physiological and biochemical parameters in living organisms. Biomolecules, such as proteins, lipids, and carbohydrates are essential for survival. Their levels change in distressed organisms, so these molecules are used as biological indicators. A change in antioxidant enzyme activity is a significant bioindicator because of the role antioxidant enzymes play in maintaining the oxidative balance in a living organism.

Selenium is an important trace element, but it also exerts significant toxic effects on insects^21^. In contrast, vitamin E is a significant nonenzymatic antioxidant because of its chain-breaking effect in the formation of free radicals^22^. Inside the body, toxic substances trigger detoxification mechanisms and undergo detoxification through biotransformation. High energy is required to produce detoxification enzymes, and heat-shock proteins play a role in this process and also actively remove toxic substances^23,24^. A decrease in the total carbohydrate and lipid content implies that the energy required for detoxification of selenium. A similar decrease in energy reserves is a result of exposure to toxic substances. This decrease has been demonstrated in different insect species^19,25^.

The observation of higher lipid and carbohydrate content in larvae fed vitamin E and selenium + vitamin E–supplemented diets in this study, compared to larvae fed a selenium-supplemented diet, is a notable finding that suggests the possible protective effects of vitamin E.

Many studies have reported that xenobiotics (e.g., pesticides and heavy metals) alter antioxidant enzyme activity and MDA content^19,26^. More recently, it has been proposed that Se-mediated thiol oxidation cause ROS^27,28^ and oxidative stress can be a factor related to Se-induced toxicity^29,30^.

Under normal physiological states, an interacting network of antioxidant enzymes effectively neutralizes the harmful effects of ROS^31^. This detoxification pathway is the result of multiple enzymes with SOD catalyzing the first step and then CAT, GPX, and various peroxidases removing hydrogen peroxide^32^.

SOD activity can increase significantly as a result of exposure to toxic metals, as shown by Li *et al*^33^. and Zhang *et al*^34^. in *Oxya chinensis* and by Emre *et al*^26^. in *G. mellonella*. Kim *et al*^3^. reported that excessive selenium induces oxidation and cross-linking of protein thiol groups and ROS generation, especially superoxide radicals. Therefore, in the present study, the increase in SOD activity is due to an increase in the superoxide radical levels caused by the selenium.

When increased CAT and SOD activity is observed together, it is believed that this increase has occurred in our study to eliminate H_2_O_2_, which is the final product formed during the elimination of superoxide radicals via increased SOD activity. Previous studies have shown a higher CAT activity related to exposure to toxic substances^33,35^. The current study presented a significantly higher CAT activity in the group fed with selenium-supplemented diet compared with the other groups. Similar increases in CAT activity caused by selenium has shown in shrimp and mouse^36,37^.

Lipid peroxidation is an important consequence of cellular damage of toxic substances^20,26,38^. Previous studies have shown that different factors, such as insecticides, heavy metals, and bacteria, cause oxidative stress and significantly increase MDA content^7,26,38^. Numerous insect species have different groups of GST enzymes^39,40^. Huang *et al*^41^. and Dubovskiy *et al*^42^. reported that GST activity increases in insects after xenobiotic (e.g., insecticide and heavy metal) exposure. In addition, selenium increases GST activity in rats^43^ and mice^44^. According to our data, depending on the increase in MDA content, the increase in GST activity might be due to an increase in lipid peroxidation products that are GST substrates^45^.

In present study, vitamin E caused a significant decrease in antioxidant enzyme activities and MDA levels compared to Se-supplemented diet. When it applied with Se, it diminished the effects of Se. Many studies have shown protective effects of vitamin E against to xenobiotic induced ROS^46–48^. Vitamin E is a well-known antioxidant, which is lipid-soluble and may block the generation of ROS^49,50^. In addition, Boldyrev *et al*^51^. reported that Vitamin E could scavenge hydroxyl radicals to protect the cells from oxidation of lipids. These decreases in antioxidant enzyme activities and MDA levels may be related to chain-breaking antioxidant activity of Vitamin E.

The circulating hemocyte count could decrease during detoxification of toxic substances^52^. Toxic substances, such as pesticides and heavy metals, decrease the number of hemocytes^19,53^. Studies have reported that toxic substances can alter the immunological response^54^, inhibit hematopoietic function^55^, decrease mitotic activity^56^, and induce apoptosis^57^. Kim *et al*^3^. and Qiao *et al*^58^. showed that high concentrations of selenium induce apoptosis, although it is a basic element. In addition, Le and Fotedar^59^ showed that high concentrations of selenium taken with food decrease the hematocrit index. A decrease in the THC might be due to induction of apoptosis or inhibition of hematopoietic function, depending on the toxic effect of selenium. This decrease in the THC can be prevented by adding vitamin E to the diet.

Low antioxidant enzyme activity and MDA content with vitamin E–supplemented diet and the positive effect of vitamin E on the THC and energy reserves might be explained by a similar inhibitory role of vitamin E on the formation of free radicals in insects and humans.

## Materials and Methods

### Test organism and experimental design

All parameters were investigated from a stock culture of *G. mellonella* larvae reared under laboratory conditions at 28 °C ± 2 °C with a relative humidity of 70% ± 5% for 24 h in the dark.

An artificial diet developed by Bronskill^60^ was used as the control diet (diet 4), which contained 58 g of bran, 3.5 g of ground honeycomb, 22 g of glycerin, 9.7 g of extracted honey, and 6.8 mL of water per 100 g of feed. Four different diets were prepared by adding 100 µg of selenium (diet 1), 100 µg of vitamin E (diet 2), and 100 µg of selenium and vitamin E each (diet 3) to the control diet. Since vitamin E was dissolved in Tween 80 and then added to the control diet, the control diet was also spiked with the same amount of Tween 80 to prepare a positive control diet (diet 5).

Second-instar *G. mellonella* larvae were obtained from the stock culture under laboratory conditions. They were fed different diets 1–5 and allowed to develop. The diets were inspected three times a day, and the developmental stages of the larvae were observed. When the larvae reached the last-instar stage (250–350 mg), feeding was stopped, and the larvae’s wet weights were recorded. The larvae were then preserved at −80 °C for biochemical tests.

### Homogenization

Four insects were homogenized in phosphate buffer (pH 7.4) and centrifuged at 24,000 rpm for 2 min. The homogenate was further centrifuged at 10,000 rpm for 30 min, and the supernatant was used to analyze enzyme activity, MDA content, and protein content.

### Determination of antioxidant enzyme activity, and MDA and protein content

The total protein content was determined as described by Lowry *et al*^61^. Albumin solution (A-2153; Sigma-Aldrich, St. Louis, MO, USA) at 1% concentration was used as a standard, and the absorbance of the supernatant was measured at a wavelength of 750 nm using a spectrophotometer. SOD activity (U/mg protein) was measured as described by Sun *et al*^62^. by indirect calculation through spectrophotometric measurements of formazone absorbance at a wavelength of 560 nm; the decrease in the optical density of formazone formed by superoxide radicals and nitroblue tetrazolium was estimated at a wavelength of 560 nm. CAT activity (U/mg protein) was measured using Aebi’s method^63^, which is based on spectrophotometric measurement of CAT that is neutralized by H_2_O_2_ per unit time at a wavelength of 240 nm. GST activity (nmol /mg protein) toward the model substrate 1-chloro-2,4-dinitrobenzene (CDNB) was assayed as described by Habig *et al*^64^. Changes in absorbance were monitored for 2 min at a wavelength of 340 nm. MDA content (nmol/mg protein) was determined using the thiobarbituric acid (TBA) method developed by Bar-Or *et al*^65^. and Dubovskiy *et al*^7^., with some modifications. Briefly, 125 μL of trichloroacetic acid (TCA) and 250 μL of the sample were mixed and centrifuged at 15,000 × *g* for 10 min. Then, 300 μL of the supernatant was mixed with 200 μL of 0.8% TBA, and the mixture was incubated at 100 °C for 60 min. Finally, absorbance was measured at a wavelength of 535 nm and MDA content determined.

### Determination of lipid and carbohydrate content

The total lipid and carbohydrate content in stock samples was determined as described by Van Handel^66,67^. Samples were homogenized in H_2_SO_4_ for 5 min at 24,000 rpm, and the lipid and carbohydrate content in the supernatant was measured. In addition, 0.1% soy oil was used as the standard for determining the lipid content. A stock solution containing 0.1 g of glucose was used to calculate the regression equation, which, in turn, was used to determine the carbohydrate content.

### Determination of the total hemocyte count

The THC/mm^3^ was calculated as described by Jones^68^. *G. mellonella* larvae were pierced on the first hind leg using a sterile needle, and 10 μL of hemolymph was withdrawn and mixed with 900 μL of Tauber–Yeager solution^69^. A drop of the diluted hemolymph was applied to a Neubauer hemocytometer. The number of hemocytes was counted under an Olympus CX21 light microscope (Olympus, Japan).

### Statistical analysis

All experiments were repeated five times each at different time points. Data between diet 4 and diets 1, 2, 3, and 5 were compared; analysis of variance was used for comparison. The Student–Newman–Keuls test was used to evaluate the significance of the difference between mean values. SPSS statistical software was used for data analysis.

## Conclusion

Selenium significantly increases antioxidant enzyme activity and MDA content and significantly decreases the primary source of energy and THC. The selected dose of selenium has a toxic effect on oxidative stress and biochemical and immunotoxic biomarkers in *G. mellonella*. Vitamin E exerts beneficial effect on inhibition of lipid peroxidation and prevention of various diseases of oxidative stress.
